# RP-HPLC Method for Simultaneous Estimation of Frusemide and Amiloride Hydrochloride in Tablet Formulation

**DOI:** 10.4103/0250-474X.70491

**Published:** 2010

**Authors:** B. P. Nagori, Renu Solanki

**Affiliations:** Lachoo Memorial College of Sciences and Technology, Pharmacy Wing, Sector-A, Shastri Nagar, Jodhpur - 342 003, India

**Keywords:** Amiloride hydrochloride, frusemide, RP-HPLC, simultaneous determination

## Abstract

A new reverse phase high performance liquid chromatography method for the simultaneous estimation of frusemide and amiloride hydrochloride in tablet formulation is developed. The determination was carried out on a HIQ SIL, C18 (250×4.6 mm, 5 µm) column using a mobile phase of 50 mM phosphate buffer solution:acetonitrile (50:50 v/v, pH 3.0). The flow rate was 1.0 ml/min with detection at 283 nm. The retention time for frusemide was 3.038 min and for amiloride hydrochloride 10.002 min. Frusemide and amiloride hydrochloride showed a linear response in the concentration range of 20-200 µg/ml and 10-100 µg/ml, respectively. The results of analysis have been validated statistically and by recovery studies. The mean recoveries found for frusemide was 99.98% and for amiloride hydrochloride was 100.09%. Developed method was found to be simple, accurate, precise and selective for simultaneous estimation of frusemide and amiloride hydrochloride in tablets.

Frusemide (FRM) is chemically 4-chloro-2-furfurylamino-5-sulphamoyl benzoic acid (fig. 1). It is a potent loop diuretic[1]. It acts primarily by blocking sodium and chloride reabsorption in the ascending limb of the loop of Henle. FRM helps to conserve potassium and minimize the risk of alkalosis, in the treatment of oedema associated with hepatic cirrhosis and congestive heart failure. Several analytical methods have been reported for quantitative determination of frusemide individually by UV[2,3], GC[4], TLC[5], HPLC[6,7] and colorimetry[8,9].

**Fig. 1:**
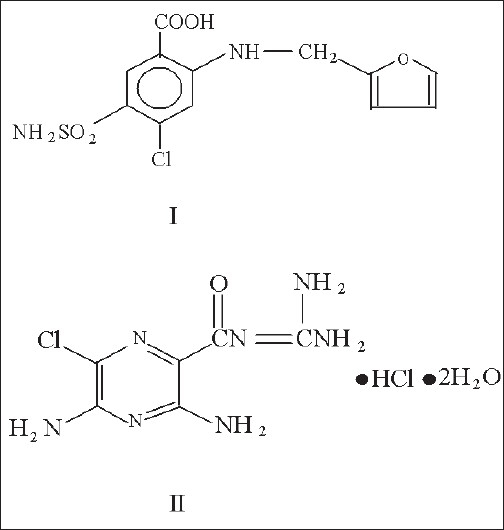
Chemical structures Chemical structures of I, frusemide and II, amiloride hydrochloride

Amiloride hydrochloride (AH) is chemically 3,5-diamino-N-(diaminomethylene)-6-chloropyrazinecarboxamide monohydrochloride dihydrate (fig. 1). It is a potassium sparing diuretic[1]. AH in conjunction with loop diuretics such as FRM, reduces overall fluid volume in the body and help to control symptoms of heart disease, kidney and liver disease[10,11]. The individual determination of AH is carried out by UV[12,13], TLC[14] and HPLC[15] methods. FRM is official in IP[16], BP[17] and USP-NF[18] and AH is official in IP[19], BP[20] and USP-NF[21].

In recent years, these two drugs are successfully used in association in the treatment of many diseases related to kidney, liver and heart and the pharmaceutical preparation containing both drugs have been marketed. Although, many methods have been reported in the literature for the estimation of FRM and AH individually, there is no single method reported for simultaneous estimation of these drugs in combined dosage form. Hence, in the present assay, a new simple, sensitive, accurate and specific reverse phase high performance liquid chromatography (RP-HPLC) method is developed and validated for simultaneous estimation of FRM and AH in tablet formulation.

Working reference standards of FRM and AH were kindly supplied as gift samples by Elder Pharmaceuticals Ltd., Mumbai, India. Two marketed formulations with brand names, Amifru (Elder Pharmaceutical Ltd., Mumbai, India) and Frumil (Geno Pharmaceuticals, Goa, India) were procured from the local pharmacy. The solvents used were of HPLC/AR grade. Double distilled water was used for analysis.

A gradient HPLC (Water, Germany) with PU-1580 double reciprocating pump, UV-1575 UV detector, and RP-C18 column (5 µm particle size) was used. The RP-HPLC system was equipped with Winchrom software for data processing. Method was developed using a HIQ SIL, C18 (250µ4.6 mm, 5 µm) column. Mobile phase was used for preparation of drug samples throughout the analysis. For preparing the mobile phase 50 mM phosphate buffer and acetonitrile were mixed together in the ratio of 50:50% v/v and pH of the resulting solution was adjusted to 3.0. It was filtered before use through 0.45 µ membrane filter. Flow rate employed was 1.0 ml/min. Detection was carried out at 283 nm at 25°.

Among the several mobile phases used for the present assay phosphate buffer and acetonitrile in the ratio of 50:50 v/v, pH 3.0 was found to be most suitable. With the above mobile phase a good resolution between FRM and AH was achieved. UV detection was carried out at 283 nm as FRM and AH both showed good absorbance at this wavelength.

Standard stock solution of FRM (200 µg/ml) was prepared by dissolving 20 mg FRM in 100 ml mobile phase. Standard stock solution of AH (100 µg/ml) was prepared by dissolving 10 mg AH in 100 ml mobile phase. Aliquots of standard stock solutions of FRM and AH were taken in 10 ml volumetric flasks and diluted upto the mark with mobile phase in such a way that final concentrations of FRM and AH were in the range of 20-200 µg/ml and 10-00 µg/ml, respectively. The standard solutions were further diluted to contain a mixture of 80 µg/ml of FRM and 10 µg/ml of AH. Twenty tablets of Amifru and Frumil each containing 40 mg of FRM and 5 mg of AH were weighed and finely powered separately. Powder equivalent to 80 mg FRM and 10 mg AH was weighed and dissolved in 100 ml mobile phase. The solution was sonicated for 15 min and was filtered through a Whatman filter paper no. 40. Further dilutions were made to get a concentration of 80 µg/ml of FRM and 10 µg/ml of AH. These solutions were filtered through 0.45 µ membrane filter. Ten microlitre solution of the each tablet was injected separately and chromatograms were recorded. A representative chromatogram is shown in fig. 2.

**Fig. 2:**
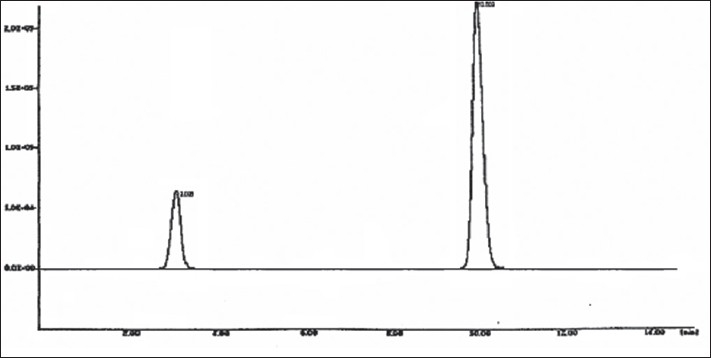
Typical chromatogram of FRM and AH. Chromatogram showing retention time, 3.038 and 10.002 for frusemide (FRM) and amiloride hydrochloride (AH) respectively

The retention time of FRM and AH was found to be 3.038 min and 10.002 min, respectively. The peak shapes of both the drugs were symmetrical and asymmetry factor was less than 2.0. The proposed method was validated as per the standard analytical procedure. Each sample was repeated 6 times and the same retention time was observed in all the cases. Linearity experiments were performed by giving six replicates for both the drugs and response was found to be linear in the range of 20-200 µg/ml of FRM and 10 -100 µg/ml of AH. Each standard solution (10 µl) was injected into the column after filtration using 0.45 µm membrane filter. The calibration curves were constructed by plotting the peak areas versus the corresponding drug concentration. The slope and correlation coefficients were determined, which were found to be 0.99995 for FRM and 0.99925 for AH. In precision studies, the injection repeatability showed a RSD of 0.069% for frusemide and 0.400% for amiloride hydrochloride. The intra-day analysis showed a RSD of 0.072% for frusemide and 0.754% for amiloride hydrochloride and the inter-day study showed a RSD of 0.024, 0.015, 0.028% for frusemide and 0.766, 0.693, 0.749% for amiloride hydrochloride for day 1, 2 and 3, respectively. These results indicate good precision of the samples analyzed. System suitability parameters of FRM and AH are given in the Table 1. Accuracy of the method was calculated by recovery studies (n=3) at five levels. Standard drug solutions containing drugs in the concentration range of 80-160 µg/ml for FRM and 10-20 µg/ml for AH were added to previously analyzed test solution containing 80 µg/ml FRM and 10 µg/ml AH. Amount of drug recovered at each level (n=3) was determined. Percent recovery at each level was calculated. The mean % recovery was found to be 99.98% for FRM and 100.09% for AH. Data from the recovery study are shown in the Table 2. The sample recovery in the marketed formulation was in good agreement with the label claim. High percentage recovery showed that the method was free from interference of excipients used in formulations. The data of result of marketed formulation analysis is shown in the Table 3. The results of the study indicate that the proposed HPLC method was simple, accurate, precise and selective. Therefore, the proposed method appears to be suitable for routine analysis of FRM and AH in their combined dosage form.

**TABLE 1:** SYSTEM SUITABILITY PARAMETERS

Parameter	Frusemide	Amiloride hydrochloride
Tailing factor*	1.04	1.01
No. of theoretical plate*	2979	9900
Asymmetry factor*	1.01	1.00
Retention time (Min.)	3.040	10.004
Resolution (R_s_)	---	6.854
Calibration Range	80-160 µg/ml	10-20 µg/ml

* Each value is the mean of 6 determinations (n=6)

**TABLE 2:** RECOVERY STUDIES WITH SAMPLESOLUTION

Drug	Amount added (µg/ml)	Recovery (%)*	Mean±SD
Frusemide	80	99.87	99.98±0.123
	100	100.20	
	120	99.99	
	140	99.92	
	160	99.94	
Amiloride hydrochloride	10	99.95	100.09±0.317
	13	99.82	
	15	100.44	
	18	100.43	
	20	99.83	

SD stands for standard deviation,

* each value is the mean of 3 determinations (n=3)

**TABLE 3:** RESULT OF MARKETED FORMULATION ANALYSIS

Marketed formulation	Drug	Label claim (mg/tab)	% mean*	±SD	SEM
Amifru (Elder Pharmaceutical Ltd.)	FRM	40	99.61	±0.243	0.140
	AH	5	95.35	±0.455	0.262
Frumil (Geno Pharmaceuticals)	FRM	40	99.78	±0.162	0.094
	AH	5	95.33	±0.507	0.292

SEM stands for standard error of the mean,

* each value is the mean of 3 determinations (n=3)
